# Ethics and moral empathy in end-of-life palliative care

**DOI:** 10.1017/S1478951525000458

**Published:** 2025-05-30

**Authors:** Ateya Megahed Ibrahim, Hassanat Ramadan Abdel-Aziz, Donia Elsaid Fathi Zaghamir, Nora H. Elneblawi, Mohamed Abd El-Rahman Elsaied Elhoty, Rasha kamal Sweelam, Heba Ahmed Osman Mohamed, Fathia gamal elsaid hassabelnaby

**Affiliations:** 1 College of Nursing, Prince Sattam Bin Abdulaziz University, Alkharj, Saudi Arabia; 2 Family and Community Health Nursing Department, Faculty of Nursing, Port Said University, Port Said, Egypt; 3 Gerontological Nursing Department, Faculty of Nursing, Zagazig University, Zagazig, Egypt; 4 Pediatric Nursing, Faculty of Nursing, Port Said University, Port Said, Egypt; 5 Department of Medical and Surgical Nursing, College of Nursing, Taibah University, Madinah, Saudi Arabia; 6 Medical Surgical Nursing, Faculty of Nursing, Helwan University, Helwan, Egypt; 7 Faculty of Nursing, Northern Border University, Arar, Saudi Arabia; 8 Psychiatric and Mental Health Nursing, Faculty of Nursing, Menoufia University, Menoufia, Egypt; 9 Maternal and Child Health Nursing Department, College of Nursing, Northern Border University, Arar, Saudi Arabia; 10 Public Health Nursing Department, Faculty of Nursing, Northern Border University, Arar, Saudi Arabia

**Keywords:** Ethics, moral empathy, palliative care, end-of-life decision-making, nursing practice

## Abstract

**Objectives:**

End-of-life care poses significant ethical challenges for nurses, requiring a deep understanding of moral empathy and ethical decision-making. This study examines the impact of these factors on end-of-life decision-making among nurses in oncology and pain management units in Egypt.

**Methods:**

A cross-sectional design was employed to gather data from participants at a single point in time, facilitating an analysis of the relationships among ethical principles, moral empathy, and nursing practice. The study involved 246 registered nurses with at least 6 months of experience, selected through stratified random sampling from oncology and pain management units in Damietta, Egypt. These settings were chosen due to their central role in palliative care, as Damietta serves as a regional healthcare hub with specialized units addressing chronic and end-of-life conditions. This selection allows for an in-depth exploration of the ethical dimensions involved in providing palliative care. Informed consent was acquired from all participants, ensuring confidentiality and the right to withdraw from the study at any time.

**Results:**

The findings indicated that 72% of participants reported high levels of moral empathy, which positively correlated with ethical decision-making scores (*r* = 0.65, *p* < 0.01). However, 58% of the nurses also reported experiencing moderate to high levels of moral distress in various clinical scenarios. Additionally, nurses in supportive ethical climates experienced significantly lower moral distress than those in less supportive settings (*p* < 0.05).

**Significance of results:**

This study highlights the importance of integrating ethical training and moral empathy into nursing education and practice. The findings underscore the need for policy reforms to embed ethics and empathy training in nursing curricula and professional development programs, fostering ethical competence and enhancing patient care quality.

## Introduction

Ethical decision-making is the foundation of end-of-life care. Nurses frequently encounter dilemmas regarding patient autonomy, beneficence, and justice. These dilemmas significantly impact decision-making processes and patient outcomes (Nikas and Green 2024; Toro 2023). Moral empathy is the main factor that helps nurses provide caring services and meet patients’ emotional and spiritual needs (Elmore et al. 2018; Isufi et al. 2024).

Palliative care involves a unique way of improving life for patients with life-threatening diseases. It is done by reducing their pain and meeting all their requirements (Ibrahim et al. 2024). Palliative care nurses have a significant role in providing compassionate care. They also help to solve the complicated ethical issues of palliative care. It can cause a nurse to experience moral distress due to balancing between the patient’s wishes and symptom control (Riera-Negre et al. 2024). The topic of nurses’ ability to make moral decisions and provide empathetic care has been one of the challenging ones in nursing education and practice (Alanazi et al. 2024).

In spite of increased recognition of ethical dilemmas in nursing, minimal research has been carried out to investigate how moral empathy influences decision-making in palliative settings, particularly in Egypt. Filling this gap is crucial for developing interventions that enhance ethical competence among nurses and consequently, the outcome of the patient (Burkhardt and Nathaniel 2024; Palmryd et al. 2024).

## Background

Providing competent end-of-life care calls for a profound comprehension of the essential principles of the nursing practice (Bollig and Zelko 2024; Gilbert and Lillekroken 2024; Keilman et al. 2024; Moureau et al. 2023). The deontological, consequentialist, and virtue-based ethical theories offer guidelines to the nurses for handling their moral dilemmas. Deontology is characterized by the fulfillment of duties and commands of moral laws, which leads to the requirement of nurses to recognize and respect the patient’s autonomy. For instance, a nurse might find him or herself asking a patient to confirm an option that denies life support even if it might harm the patient. The case is based on end-of-life care. A real-world example is a nurse who does not disagree with a wish of a dying patient about no further action to be taken even if the nurse knows that this action would prolong the life of the patient.

Conversely, consequentialism concentrates on the results of actions prompting nurses to consider the potential consequences of decisions on patients’ welfare. Thus, a nurse can opt to administer treatment if it can extend a patient’s life even if the patient has not communicated his/her wishes. For instance, a nurse can choose to administer treatment if it is capable of extending the patient’s life knowing that the patient will have more time to make a decision or say goodbye to relatives.

Virtue ethics, in contrast, focuses on the moral character of the agent, which in this case are the nurses, who are called upon to develop virtues such as compassion and integrity in their practice. As a result, the nurse may create a comfortable and dignified end-of-life experience, showing compassion in the face of the fact that the medical procedures are no longer beneficial. For instance, in case there is no benefit in the use of medical procedures, the nurse would abandon them and focus on alleviating the pain and ensuring the comfort of the patient, which is an indication of both compassion and respect for the dignity of the patient (Fowler 2024).

Moral empathy is crucial in the development of compassionate care. The empathetic nurse is likely to attend effectively to the needs of the patients, hence increasing patient satisfaction (Fowler 2024). For example, if a patient is suffering from a fear of dying; the empathetic nurse can easily comfort the patient by holding his or her hand and talking to him or her. The patient will feel relieved, and thus the nurse will have given emotional support. Sometimes ethical dilemma arises, and the nurses are torn between the idea of autonomy, beneficence, and justice. Nurses are also expected to balance their professional duties with the values and preferences of the patient and his or her family. For instance, the decision to withhold or withdraw the life-sustaining treatment can cause moral distress because the nurse must consider the autonomy of the patient and the preference of his or her family. The ethical climate in nursing is important in addressing moral distress and examining the ethical climate in nursing practice. The ethical climate in healthcare organizations significantly impacts nurses’ ability to engage in moral reasoning and empathetic care. An ethical climate that is supportive fosters open communication and collaboration, enabling nurses to advocate for patients effectively (Gilbert and Lillekroken 2024; Keilman et al. 2024). Conversely, an ethical climate that is unsupportive may lead to moral distress and burnout, complicating the delivery of empathetic care (Fowler, 2024).

## Significance of the study

This study has significant implications for nursing practice, education, and research. The examination of the role of ethics and moral empathy in end-of-life care will provide a wealth of information regarding how they affect the decision-making process and patient experiences. The results of the research will help in developing educational programs that will enable nurses to handle ethical issues well, thus improving the quality of care and patient outcomes. The findings will also contribute to the development of hospital policies, nursing curricula, and professional guidelines that will reduce moral distress and enhance ethical decision-making.

## Theoretical framework

The theoretical framework of this study incorporated several fundamental ethical theories and models that support moral reasoning in the nursing practice. Specifically, the end-of-life care.

## Deontological ethics

Deontological ethics theory as posed by Beauchamp and Childress highlights nurse’s commitment to moral rules, and ensures respect of patient autonomy, and integrity in care. This theory allows nurses to make decisions based on duties rather than the outcomes of actions (Burkhardt and Nathaniel 2024).

## Consequentialism

Complementing this is consequentialism, which instructs nurses to weigh the outcomes of their actions and prioritize the well-being and overall impact on the quality of life of patients. This perspective concentrates on the evaluation of potential benefits and harms of actions in the context of patient care (Selvakumar and Kenny 2023).

## Virtue ethics

Virtue ethics is concentrated on the character and moral progress of the nurse, recommending that virtues such as compassion, empathy, and integrity be developed to guide ethical practice. The theory emphasizes the importance of the nurse’s personal characteristics in making ethical decisions in palliative care (Rushton 2024).

## Ethic of care theory

To further enrich this framework, the theory of care ethics emphasizes the importance of the relations and empathy between them, reflecting the moral significance of establishing caring relations with patients. This theory supports the idea that moral decisions in healthcare are not only about rules or outcomes, but about the quality of relationships with patients (Benbow et al. 2024).

## Moral distress theory

Moral distress theory is also essential as it focuses on the emotional and ethical stress that nurses experience when they cannot act according to their ethical beliefs, especially in palliative care settings. This theory brings into focus the challenges that nurses face in aligning their ethical beliefs with the actual care delivery (Fowler 2024).

## Conceptual framework

The study’s guiding conceptual framework embraces and integrates various ethical theories with the psychological constructs stressing the interconnectedness of moral reasoning, emotional intelligence, and nursing professional behavior. Ethical principles such as autonomy (decision-making by patients about the care they will receive), beneficence (the obligation to sustain the well-being of a patient), non-maleficence (the obligation not to cause harm), and justice (fairness and equity in the distribution of health interventions) form a sturdy foundation upon which decisions in palliative care can be made (Burkhardt and Nathaniel 2024).

On the contrary, moral empathy is the view, understanding, and responding cognitively and emotionally to the emotional states of others, particularly under life-threatening and life-changing conditions in dealing with patients. This type of empathy enhances the nurse’s ability to approach ethically troubling situations with a balanced head and heart so that decisions about patient care are assumed to be sensitive to the backgrounds of patients and their families involved emotionally (Rushton 2024; Selvakumar and Kenny 2023).

The framework further embraces emotional intelligence as a significant component that affects ethical behavior of nursing. Emotional intelligence supports nurses in controlling their responses to difficult situations, promoting communication and empathy in complex patient scenarios. In addition, emotionally intelligent nurses are better positioned to manage ethical dilemmas, maintaining a balance between patient autonomy and family interests, while ensuring that all views are recognized and considered in the decision-making process (Benbow et al. 2024).

The framework recognizes that end-of-life decision-making implicates not only ethical principles but also ethical dilemmas, in which conflicting values need to be carefully balanced (Burkhardt and Nathaniel 2024; Selvakumar and Kenny 2023). By incorporating these ethical principles and psychological constructs, the framework promotes a comprehensive approach to palliative care, in which care decisions are based on a well-informed ethical responsibility and empathy.

## Aim of the study

To examine the impact of ethics and moral empathy on end-of-life care in palliative settings, with the goal of improving nursing practices and patient outcomes in palliative care environments.

## Design

This study used a cross-sectional design in assessing the impact of ethics and moral empathy on palliative care at life’s end. The design gave a snapshot of observation at a specific point in time that made it simple to carry out a complete analysis of research variable relationship.

## Settings

The study was carried out in the oncology and pain management units of one government hospital in Damietta, Egypt. These units were chosen because they deal with palliative care in the form of managing chronic diseases and providing end-of-life care for patients with terminal diseases. The oncology unit has around 30 beds and provides multidisciplinary cancer care. The pain management unit provides care for patients with severe pain from chronic diseases and terminal conditions. The 2 units provide an ideal area for researching the ethical issues in care and the role of moral empathy in decision-making. The units are the major providers of palliative care, which is the focus of the study.

## Sample size and sampling technique

The sample size for this study was carefully calculated using G*Power software to ensure adequate statistical power. With a medium effect size (Cohen’s *d* = 0.5), a significance level of 0.05, and a power of 0.80, the study required a total of 246 participants. This sample size ensures the ability to detect medium-effect size relationships between ethical decision-making and moral empathy in palliative care settings. A stratified random sampling technique was employed to ensure that the sample was both diverse and representative of nurses in oncology and pain management units. The following factors were used for stratification:

- Department: Nurses were stratified by department – oncology and pain management – to capture potential differences in ethical decision-making and moral empathy based on the nature of the care provided in each unit.
- Experience level: Nurses were grouped by years of experience in palliative care (less than 5 years, 5–10 years, and over 10 years). This stratification was intended to explore how varying levels of experience influence the nurses’ ethical decision-making processes and empathy.
- Shift: Nurses were also stratified by the shift they worked (day, evening, or night) to account for any potential differences in work conditions and patient care needs that might impact their moral reasoning and empathetic responses. These stratification categories were carefully selected because they are likely to influence ethical challenges and decision-making in palliative care, making them important factors to include in the study.

The use of stratified random sampling not only enhances the diversity and balance of the sample but also ensures that nurses from different backgrounds and work environments are adequately represented. By including nurses from various experience levels, shifts, and departments, the study aims to capture a broad range of perspectives, making the findings more generalizable to the wider nursing community in Egypt. Additionally, this approach helps mitigate selection bias and provides a comprehensive understanding of how ethical decision-making and moral empathy manifest across different settings and contexts in palliative care.

## Inclusion and exclusion criteria

Inclusion criteria for the study consisted of registered nurses in Egypt who were directly involved in patient care within the oncology and pain units. Participants were required to have a minimum of 6 months of experience in their current settings to ensure familiarity with the ethical challenges faced in end-of-life care. Exclusion criteria encompassed nurses not directly involved in patient care (e.g., administrative staff and educators) and those who had less than 6 months of experience or were primarily assigned to inpatient units.

## Recruitment process

Recruitment of the participants included direct outreach, email invitations, and informational posters in the oncology and pain management units. Registered nurses, who showed interest, received detailed information on the study’s purpose, procedures, and potential risks and benefits. There were informational posters on the significance of the research and contact information for interested participants. All participants had to sign the informed consent form before taking part in the study. The recruitment process was designed to provide diversity within the sample, which would give a more comprehensive understanding of the ethics and moral empathy in end-of-life care. To avoid selection bias, nurses from different shifts and roles in the oncology and pain management units were recruited.

## Tools of data collection

### Interpersonal Reactivity Index

Interpersonal Reactivity Index (IRI) developed by Davis (1983) assesses different dimensions of individual differences in empathy and is therefore suited for clinical and caregiving settings. It has 4 subscales: Perspective Taking, Empathic Concern, Personal Distress, and Fantasy. Each subscale focuses on a different facet of empathy: the examination of how an individual takes another’s viewpoint, feels compassion for him or her, experiences the distress caused by another’s emotion, or identifies with a fictional character. This multidimensional approach is highly relevant in a health-related view, where emotional sensitivity is a crucial aspect. The subjects were asked to indicate their level of agreement with each item on a 5-point Likert scale, with higher scores indicating more empathy.

### Moral Distress Scale

The Moral Distress Scale (MDS), developed by Corley et al. (2005), measures the level of moral distress experienced by healthcare professionals when faced with ethical dilemmas, especially in situations constrained by organizational policies or patient care decisions. It assesses both patient-related and system-related sources of distress, providing insights into the frequency of moral conflict and frustration in practice. Each item is rated on a scale from 0 (not at all) to 4 (very frequently), with higher scores indicating more frequent experiences of moral distress. This tool is instrumental in identifying moral distress triggers and their impacts on healthcare workers’ well-being.

### Ethical Climate Index

The Ethical Climate Index (ECI), developed by Victor and Cullen (1988), assesses the ethical climate within organizations, focusing on how healthcare professionals perceive their ethical environment. The ECI covers dimensions such as caring, law and code, rules and procedures, and self-interest, offering insights into how ethical issues are addressed in the workplace. Respondents rate items on a Likert scale, with higher scores in particular dimensions reflecting a stronger presence of that ethical aspect within the organization. This tool is especially useful in healthcare settings, where ethical decision-making is central to patient care, helping to identify whether the organizational climate supports ethical practices.

### Palliative Care Outcome Scale

The Palliative Care Outcome Scale (POS), developed by Hearn and Higginson (1999), evaluates the effectiveness of palliative care interventions by focusing on dimensions such as symptom management, psychological well-being, spirituality, and family impact. It provides a holistic overview of the patient’s quality of life, assessing both physical and emotional components of care in palliative settings. The POS includes items scored on a 5-point scale, with higher scores indicating poorer outcomes. It is widely used in research to measure changes in patients’ conditions following palliative care interventions.

## Validity and reliability

In ensuring quantitative evidence about the validity and reliability of the instruments used in the study, construct validity was confirmed through confirmatory factor analysis. The analysis showed that the items in each scale loaded well on their respective factors, thereby confirming the theoretical underpinning of the IRI, MDS, ECI, and POS. Content validity, on the other hand, was established through the review of the instruments by a panel of experts, ensuring that the instruments measured what they were intended to measure. For a high degree of reliability, Cronbach’s alpha was computed for each of the scales and revealed values of 0.88 for IRI, 0.85 for MDS, 0.82 for ECI, and 0.90 for POS, indicating an excellent internal consistency. It can then be concluded that the reliability coefficients support the fact that the instruments can be claimed valid and consistent in measuring the constructs in the sample population, which ultimately increases the trustworthiness of the study’s findings.

## Ethical considerations

Ethical approval for this study was obtained from the Faculty of Nursing, Zagazig University (ID/Zu.Nur.REC:00269). The study adhered to ethical guidelines established by the Declaration of Helsinki, ensuring the protection of participants’ rights and welfare. Informed consent was obtained from all participants before their involvement in the study, assuring them that their participation was voluntary and that they could withdraw at any time without any consequences. Confidentiality was maintained throughout the research process by anonymizing and securely storing data. The research team was committed to upholding ethical standards to promote integrity and respect in the conduct of this study.

## Statistical analysis

The statistical analyses for this study were conducted using Statistical Package for the Social Sciences version 28. Descriptive statistics were calculated to summarize the key variables, including means and standard deviations for the IRI, MDS, ECI, and POS. To explore relationships between the variables, Pearson correlation coefficients were computed, identifying significant correlations among empathy, moral distress, and palliative care outcomes. ANOVA was employed to assess differences in moral distress across ethical climate groups, followed by post hoc comparisons to pinpoint specific group differences. Additionally, the Kruskal–Wallis test was utilized to evaluate variations in POS scores by moral distress levels, given the non-normally distributed nature of the data. Finally, multiple regression analysis was performed to examine the predictive relationships between empathy dimensions and moral distress, providing insights into how these factors influence one another within the context of end-of-life palliative care.

## Results

Table 1 presents descriptive statistics for the key variables measured in the study. The IRI reveals that participants reported a mean score of 3.25 for Perspective Taking and 3.40 for Empathic Concern, indicating generally high levels of empathy. Conversely, Personal Distress had a lower mean of 2.10, suggesting less self-reported distress in response to others’ suffering. The MDS indicated a mean score of 2.75 for Patient-related Distress and 2.50 for System-related Distress, reflecting moderate moral distress levels among participants. The ECI showed that Caring received the highest mean score (3.50), followed by Law and Code (3.20), suggesting a positive ethical climate. Lastly, the POS indicated mean scores of 3.75 for Symptoms and 3.40 for Psychological outcomes, while Spirituality scored lower at 2.80.

**Table 1.** Descriptive statistics of study variables

Variable	Mean (SD)
Interpersonal Reactivity Index (IRI)	
Perspective Taking	3.25 (0.56)
Empathic Concern	3.40 (0.61)
Personal Distress	2.10 (0.74)
Fantasy	2.85 (0.68)
Moral Distress Scale (MDS)	
Patient-related Distress	2.75 (0.73)
System-related Distress	2.50 (0.78)
Ethical Climate Index (ECI)	
Caring	3.50 (0.49)
Law and Code	3.20 (0.61)
Rules and Procedures	2.90 (0.72)
Self-Interest	2.40 (0.83)
Palliative Care Outcome Scale (POS)	
Symptoms	3.75 (0.80)
Psychological	3.40 (0.75)
Spirituality	2.80 (0.65)
Family	3.10 (0.70)

In Table 2, the correlation matrix reveals significant relationships among the variables. A strong positive correlation exists between Empathic Concern and Perspective Taking (*r* = 0.68), emphasizing the interrelatedness of these aspects of empathy. Conversely, both forms of moral distress (patient-related and system-related) showed negative correlations with empathy metrics, indicating that higher empathy is associated with lower levels of distress. Notably, Symptoms from the POS had a strong negative correlation with both IRI measures and a positive correlation with MDS scores, suggesting that greater symptoms correlate with higher moral distress.

**Table 2.** Correlation matrix of key variables

Variable	IRI PT	IRI EC	MDS PD	MDS SD	ECI Caring	ECI Law and Code	POS Symptoms	POS Psychological	POS Spirituality
IRI – Perspective Taking (PT)	1								
IRI – Empathic Concern (EC)	0.68**	1							
MDS – Patient-related Distress (PD)	−0.45**	−0.40**	1						
MDS – System-related Distress (SD)	−0.32**	−0.38**	0.58**	1					
ECI – Caring	0.50**	0.52**	−0.45**	−0.35**	1				
ECI – Law and Code	0.38**	0.45**	−0.42**	−0.28**	0.50**	1			
POS – Symptoms	−0.55**	−0.48**	0.60**	0.40**	−0.55**	−0.48**	1		
POS – Psychological	−0.48**	−0.55**	0.45**	0.38**	−0.50**	−0.42**	0.58**	1	
POS – Spirituality	−0.30**	−0.25**	0.30**	0.28**	−0.35**	−0.25**	0.38**	0.30**	1

** *p* < 0.01 this indicates a statistically significant correlation at the 0.01 level.

ECI = Ethical Climate Index; IRI = Interpersonal Reactivity Index; MDS = Moral Distress Scale; POS = Palliative Care Outcome Scale.

The ANOVA results displayed in Table 3 indicate significant differences in moral distress levels across ethical climate groups. Specifically, the High Caring group reported significantly lower scores on both dimensions of the MDS (2.25 for Patient-related Distress and 2.00 for System-related Distress) compared to the Low Caring group (3.25 and 3.00, respectively). These findings underscore the influence of an ethical climate characterized by care on reducing moral distress.

**Table 3.** ANOVA results for moral distress by ethical climate groups

Ethical climate group	Mean MDS PD (SD)	Mean MDS SD (SD)	*F*(2, 243)	*p*-value
High Caring	2.25 (0.66)	2.00 (0.71)	6.15	<0.01
Moderate Caring	2.80 (0.72)	2.50 (0.74)		
Low Caring	3.25 (0.80)	3.00 (0.79)		

Table 4 presents the results from the Kruskal–Wallis test, revealing significant differences in POS scores across moral distress levels. Participants with Low Moral Distress reported a median score of 2.50 for Symptoms, while those with High Moral Distress reported a median score of 4.00, indicating that lower moral distress is associated with better palliative care outcomes. Similar trends are evident for Psychological and Spirituality scores, highlighting the detrimental impact of moral distress on overall outcomes.

**Table 4.** Kruskal–Wallis test for POS scores by moral distress levels

Moral distress level	Median POS Symptoms	Median POS Psychological	Median POS Spirituality	*H*(2)	*p*-value
Low Moral Distress	2.50	2.75	2.40	7.15	<0.05
Moderate Moral Distress	3.10	3.30	2.80		
High Moral Distress	4.00	3.80	3.00		

Regression analysis in Table 5 highlights the predictive relationship between empathy and moral distress. The model reveals that both Perspective Taking (*β* = −0.30) and Empathic Concern (*β* = −0.28) are significant predictors of reduced moral distress, while Personal Distress is a positive predictor (*β* = 0.45). The model explains 42% of the variance in moral distress, emphasizing the importance of empathy in mitigating distress among healthcare providers.

**Table 5.** Regression analysis of empathy on moral distress

Predictor variable	*B*	SE	*β*	*t*	*p*-value
Constant	4.20	0.50	-	8.40	<0.01
IRI – Perspective Taking	−0.40	0.12	−0.30	−3.33	<0.01
IRI – Empathic Concern	−0.35	0.11	−0.28	−3.18	<0.01
IRI – Personal Distress	0.50	0.10	0.45	5.00	<0.01

*R*^2^ = 0.42; adjusted *R*^2^ = 0.40.

Lastly, Table 6 summarizes the palliative care outcomes stratified by ethical climate and moral distress levels. High Caring settings demonstrated significantly better mean scores for Symptoms, Psychological, and Spirituality compared to Low Caring environments. This finding further supports the notion that an ethical climate fostering care can enhance palliative care outcomes while reducing the impact of moral distress on healthcare providers.

**Table 6.** Summary of palliative care outcomes by ethical climate and moral distress

Outcome variable	Ethical climate (high/low)	Mean scores (SD)	*p*-value
Symptoms	High Caring	2.50 (0.70)	<0.01
Symptoms	Low Caring	4.00 (0.75)	
Psychological	High Caring	3.20 (0.70)	<0.05
Psychological	Low Caring	3.80 (0.68)	
Spirituality	High Caring	2.60 (0.70)	<0.05
Spirituality	Low Caring	3.20 (0.65)	

## Post hoc comparison

- High Caring vs. Low Caring: *p* < 0.05
- Moderate Caring vs. Low Caring: *p* < 0.05

## Discussion

The descriptive statistics revealed significant insights into the characteristics of the study participants, particularly their empathy, moral distress, ethical climate, and palliative care outcomes. The variability in scores on the IRI suggested that individual differences in empathy exist among nurses. This finding aligns with previous research, demonstrating that variability in empathy levels can significantly influence patient interactions (Babaii et al. 2021; Hovland et al. 2021; Juniarta and Ferawati Sitanggang 2024; Vujanić et al. 2022). Additionally, the moral distress scores indicated that a substantial number of nurses experience ethical dilemmas, supported by studies highlighting that moral distress arises when nurses feel unable to act according to their ethical beliefs due to various constraints (Alhaddad, 2024; Aljabery et al. 2024; Ghazanfari et al. 2022; Prompahakul et al. 2021; Salas-Bergüés et al. 2024). Thus, the observed levels of moral distress underscore the pressing need for interventions aimed at enhancing ethical practices within clinical settings.

The correlation matrix illustrated the relationships among key variables, demonstrating that higher empathy correlates with lower moral distress. This finding is supported by studies indicating that empathy serves as a protective factor against moral distress. When nurses empathize with their patients, they may better navigate ethical challenges, leading to reduced feelings of distress (Helmers et al. 2020; Lamiani et al. 2020; Morley et al. 2021; Prignano et al. 2024). Furthermore, the positive correlation between ethical climate and palliative care outcomes suggests that a supportive ethical environment fosters better patient care. This is consistent with findings from other studies arguing that organizations should cultivate an ethical climate to enhance both nurse well-being and patient outcomes (Al’Ararah et al. 2024; Kohnen et al. 2024; Koskenvuori et al. 2019; Rushton et al. 2023).

The ANOVA results indicated significant differences in moral distress levels based on the ethical climate of the workplace. Nurses in environments that promote ethical practices report lower levels of moral distress, consistent with studies finding that supportive ethical climates reduce moral distress (Borrelli et al. 2024; Epstein et al. 2023; Helmers et al. 2020; Morley et al. 2021 ). Thus, the results emphasize that healthcare organizations must prioritize ethical practices and create environments where nurses feel empowered to make ethically sound decisions, ultimately leading to enhanced nurse satisfaction and patient care.

The Kruskal–Wallis test results indicated that higher levels of moral distress correlate with poorer palliative care outcomes. Researchers emphasize that when nurses experience moral distress, their ability to provide quality care may be compromised. This finding is supported by studies noting that moral distress negatively impacts patient interactions and care quality (Corradi-Perini et al. 2021; De Brasi et al. 2021; Deschenes et al. 2020; Morley et al. 2022). Therefore, addressing moral distress is critical for improving patient outcomes in palliative care settings. Researchers advocate for developing support systems that help nurses cope with ethical dilemmas, thereby enhancing their capacity to provide compassionate care.

The regression analysis demonstrates that specific dimensions of empathy, particularly empathic concern, significantly predict levels of moral distress among nurses. This finding is supported by studies highlighting that fostering empathy can mitigate moral distress. Higher empathic concern enables nurses to connect with patients on a deeper level, potentially reducing feelings of helplessness in ethical situations (Morley et al. 2021). Researchers suggest that training programs aimed at enhancing empathy could be beneficial for reducing moral distress, ultimately promoting better patient care.

The summary of palliative care outcomes shows that both ethical climate and moral distress influence the quality of care delivered. Nurses in positive ethical climates report significantly better patient outcomes, reinforcing the argument that ethical environments enhance care effectiveness. This finding is supported by various studies emphasizing the importance of ethical practices in healthcare settings (Corradi-Perini et al. 2021; De Brasi et al. 2021; Deschenes et al. 2020; Morley et al. 2022). Researchers argue that organizations must focus on cultivating an ethical climate to improve the overall quality of care in palliative settings, benefiting both patients and enhancing the job satisfaction of nursing staff.

## Conclusion of the study

The study explored the intricate relationships between ethics, moral empathy, and decision-making in end-of-life palliative care among nurses. The findings indicate that higher levels of moral empathy are associated with improved ethical decision-making and reduced moral distress, highlighting the critical role of emotional intelligence in nursing practice. By understanding the ethical dimensions of care and fostering moral empathy, healthcare professionals can enhance patient outcomes and support families more effectively during challenging times. Overall, this study underscores the necessity of integrating ethical training and empathy development into nursing education and practice.

## Recommendations

Based on the findings, it is recommended that nursing education programs incorporate comprehensive training on ethical principles and moral empathy to better prepare future nurses for the complexities of end-of-life care. Additionally, healthcare organizations should implement support systems that foster an ethical climate, allowing nurses to express their moral concerns without fear of repercussions. Workshops and seminars on empathy training and ethical decision-making can further equip nurses to navigate the emotional challenges inherent in palliative care settings, ultimately benefiting both patients and healthcare providers.

## Study implications

The implications of this study extend beyond individual nursing practice to influence healthcare policy and organizational culture. By emphasizing the importance of ethical principles and moral empathy, healthcare institutions can cultivate a more supportive environment for nurses, which can enhance job satisfaction and reduce burnout. Additionally, fostering a work environment that prioritizes ethical decision-making and emotional well-being can improve retention rates and overall job satisfaction among nursing staff. Integrating these concepts into clinical guidelines may lead to improved patient care and outcomes in palliative settings, reinforcing the importance of addressing both ethical and emotional dimensions in healthcare practices. Furthermore, healthcare organizations can implement training programs to better equip nurses in managing moral distress and ethical dilemmas, ultimately leading to better decision-making at the point of care and a more compassionate approach to end-of-life care.

## Study strengths

This study demonstrates several strengths that contribute to its overall validity and relevance in the field of nursing and palliative care. First, the use of well-established measurement tools, such as the IRI and the MDS, ensures that the assessment of key variables – moral empathy and ethical decision-making – is reliable and grounded in empirical research. Additionally, the cross-sectional design allows for the exploration of relationships among variables at a single point in time, providing insights into current practices and attitudes among nurses in palliative settings. The focus on oncology and pain management units enhances the specificity of the findings, making them particularly relevant for understanding the ethical challenges faced in these critical areas of care. Furthermore, the study’s implications for nursing education and practice highlight the importance of addressing ethical and emotional dimensions, which can inform future training and support initiatives, ultimately improving patient care and nurse well-being.

## Study limitations

Despite its contributions, this study has several limitations. The cross-sectional design restricts the ability to draw causal conclusions about the relationships between moral empathy, ethical decision-making, and moral distress. Additionally, the study was conducted in a specific geographical location, which may limit the generalizability of the findings to other contexts or cultures. The reliance on self-reported measures may also introduce bias, as participants may respond in socially desirable ways. Future research should consider longitudinal designs and diverse settings to better understand these relationships over time.
